# Supplementary material for: Detection and Characterization of Leptospira Infection and Exposure in Rats on the Caribbean Island of Saint Kitts
Source: Animals (Basel). 2020 Feb 22;10(2):350. doi: 10.3390/ani10020350 (PMC7071179; doi:10.3390/ani10020350)
Supplement: Supplementary file 1 [file animals-10-00350-s001.pdf]

Supplementary Material

Detection and characterization of *Leptospira* infection and exposure in rats in the Caribbean island of Saint Kitts

Sreekumari Rajeev, Kanae Shiokawa, Alejandro Llanes, Malavika Rajeev, Carlos Mario Restrepo, Raymond Chin, Eymi Michelle Cedeño and Esteban Ellis

Table S1. General features of sequenced genomes and genetic variants for *Leptospira* isolates.

Isolate	Species[a]	Host	Serogroup	Size (Mb)	GC%	Total number of variants[b]	Variants within coding sequences[b]	Genbank accession[c]
R6	LB	RR	Ballum	4.1	40.0	166	97	CP047372 CP047373
R6L	LB	RR	Ballum	4.1	40.1	166	97	CP047520 CP047521
R7	LI	RN	Ictero	4.7	35.1	194	38	CP047512 CP047513
R11	LI	RN	Ictero	4.7	35.1	200	41	CP047510 CP047511
R12	LI	RN	Ictero	4.7	35.1	192	39	CP047508 CP047509
R13	LI	RN	Ictero	4.7	35.1	204	43	CP047506 CP047507
R13L	LI	RN	Ictero	4.7	35.1	195	43	CP047518 CP047519
R14	LB	RN	Ballum	4.0	40.1	156	90	CP047504 CP047505
R14L	LB	RN	Ballum	4.0	40.0	157	91	CP047516 CP047517
R16	LI	RN	Ictero	4.7	35.1	203	42	CP047502 CP047503
R17	LI	RN	Ictero	4.7	35.2	202	44	CP047500 CP047501
R19	LI	RN	Ictero	4.7	35.1	201	43	CP047514 CP047515
R21	LI	RN	Ictero	4.7	35.2	202	42	CP047498 CP047499
R22	LI	RN	Ictero	4.7	35.1	198	41	CP047496 CP047497
R23	LB	RR	Ballum	4.0	40.0	155	90	CP047370 CP047371
R28	LB	RR	Ballum	4.1	40.0	164	93	CP047332 CP047333
R29	LB	RR	Ballum	4.1	40.0	158	90	CP047330, CP047331

[a] For isolates with evidence of mixed infection, only the genome for the species with the higher percentage of reads in the sample was considered. [b] Including both single nucleotide polymorphisms (SNP) and small insertion/deletions (indels) up to 3 bp. [c] Chromosomes I and II, in that order. LI = *L. interrogans*; LB = *L. borgpetersenii*; MM = *M. musculus*; RN = *R. norvegicus*; RR = *R. rattus*; Ictero = Icterohaemorrhagiae

Table S2. Test results from individual animals, including collection date and site.

											MAT Titers				
ID	Spp	Date	Site[1]	Sex	Age	DFA	RT-PCR	C_t value	Culture	MAT results	Mankarso	Ictero	Copenhageni	Grippotyphosa	Wolffi
R1	RN	03/04/17	B	F	M	0	1	30.21	0	1	50	0	0	0	0
R2	RR	03/07/17	A	F	M	0	0	40	0	1	0	50	0	0	0
R3	RR	03/07/17	A	M	J	0	1	38.49	0	1	0	50	0	0	0
R4	RR	03/07/17	A	M	J	0	0	40	0	1	0	50	0	0	0
R5	RR	03/08/17	A	M	M	0	0	40	0	1	0	50	0	0	0
R6	RR	03/08/17	A	M	M	0	1	36.03	1	0	0	0	0	0	0
R7	RN	03/14/17	B	F	M	1	1	29.94	1	1	0	400	200	0	0
R8	RN	03/17/17	B	F	J	0	0	40	0	0	0	0	0	0	0
R9	RR	03/18/17	D	F	M	0	0	40	0	1	0	50	0	0	0
R10	RN	03/25/17	B	F	M	0	0	40	0	1	0	200	0	0	0
R11	RN	04/03/17	C	M	M	1	1	21.78	1	1	0	200	200	0	0
R12	RN	04/03/17	C	M	M	1	1	32.34	1	1	0	50	0	0	0
R13	RN	04/03/17	C	F	M	1	1	19.32	1	1	0	1600	0	0	0
R14	RN	04/04/17	C	M	M	1	1	18.11	1	1	0	1600	200	0	0
R15	RR	04/04/17	C	M	M	0	0	40	0	1	0	50	0	0	0
R16	RN	04/06/17	C	M	M	1	1	21.23	1	1	0	800	200	0	0
R17	RN	04/07/17	C	F	M	1	1	28.64	1	1	0	100	0	0	0
R18	RN	04/07/17	C	M	J	0	0	40	0	1	0	50	0	0	0
R19	RN	04/07/17	C	F	M	1	1	26.13	1	1	0	100	0	0	0
R20	RN	04/12/17	C	F	M	0	0	40	0	0	0	0	0	0	0
R21	RN	04/12/17	C	M	J	0	0	40	1	1	0	400	0	50	0
R22	RN	04/13/17	C	F	M	1	1	29.11	1	1	0	200	0	0	0
R23	RR	04/14/17	C	F	M	0	1	31.61	1	1	50	200	0	50	100
R24	RR	04/14/17	C	F	M	0	0	40	0	1	0	50	0	0	0
R25	RN	04/25/17	C	M	M	0	1	28.79	0	1	0	200	0	0	0
R26	RN	04/25/17	C	M	M	0	0	40	0	0	0	0	0	0	0
R27	RN	04/26/17	C	M	M	0	1	38.99	0	1	0	50	0	0	0
R28	RR	04/27/17	C	F	M	1	1	21.24	1	1	50	100	0	0	0
R29	RR	04/27/17	C	M	M	1	1	26.94	1	1	50	200	0	0	0

[1] Sites according labels from Figure S1. Species: RN = *Rattus norvegicus*, RR = *Rattus rattus*;
Age: M = Mature, J = Juvenile; Ictero: Icterohaemorrhagiae; 0 = Negative, 1= positive;
Ct value: Cycle threshold for PCR

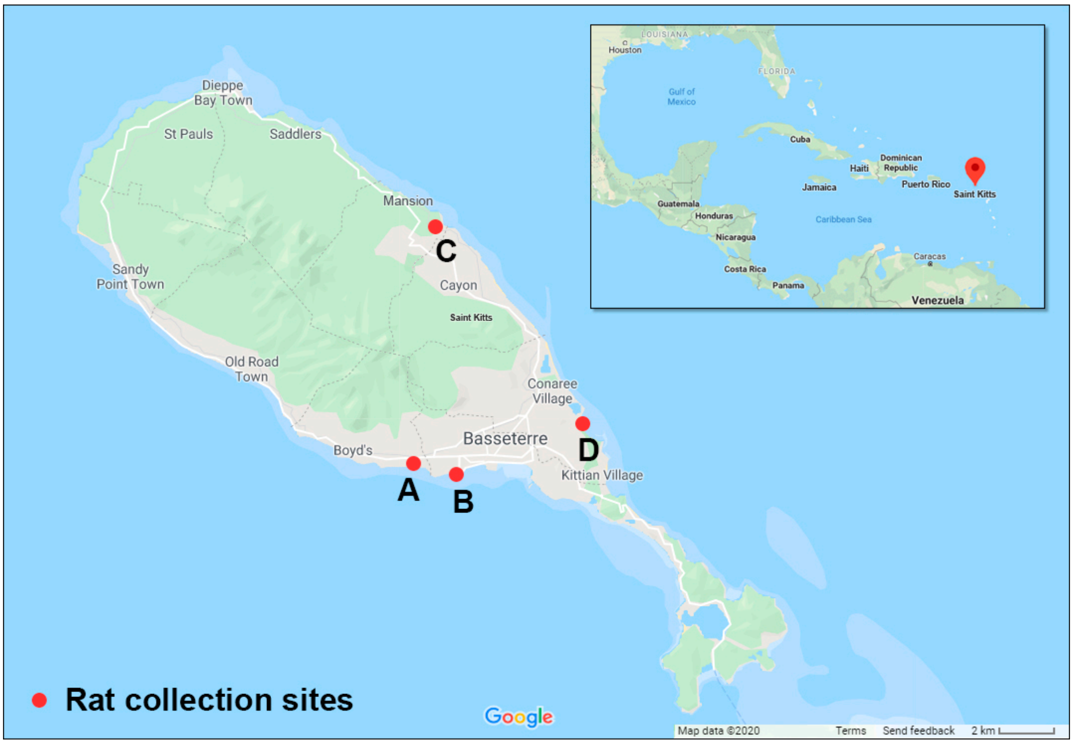

Figure S1. Geographic location of rat collection sites in the island of Saint Kitts. A – Esteridge, B – Basseterre, C – Dewars State, D – Half Moon Bay.

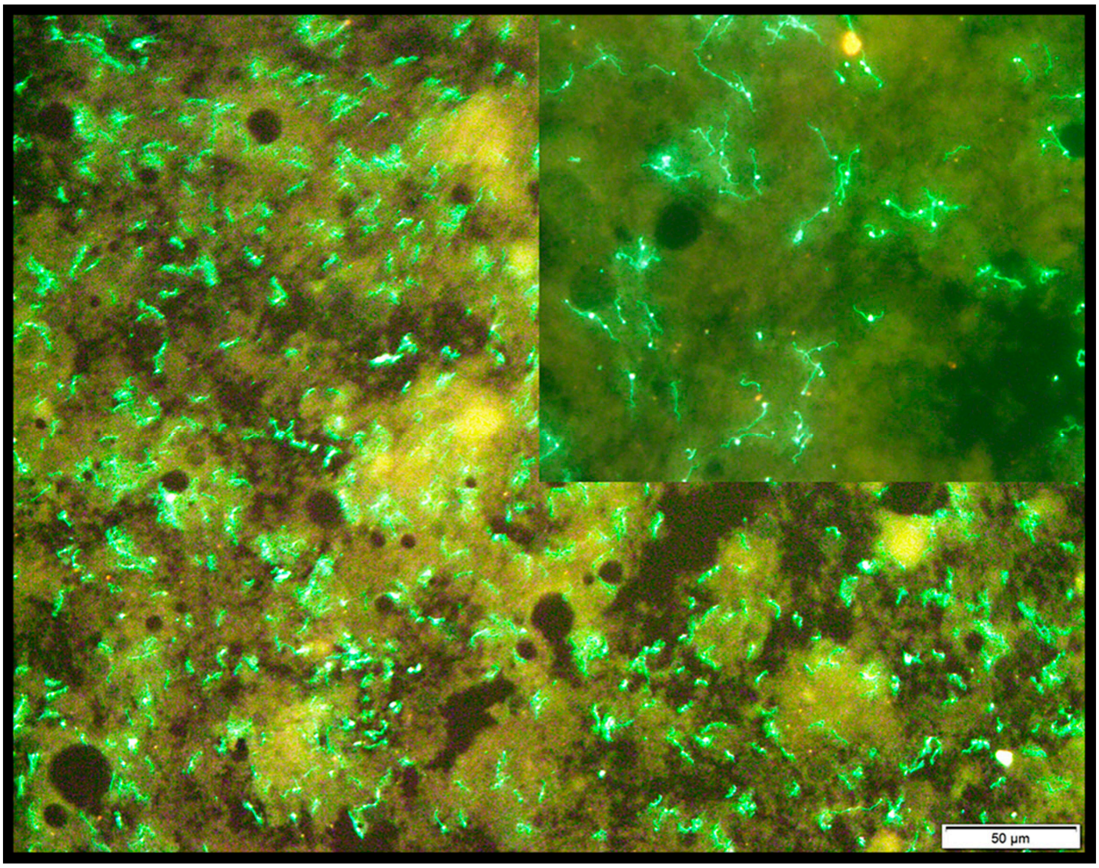

Figure S2. Direct immunofluorescence assay (DFA) of a representative rat kidney. Main figure shows numerous fluorescent organisms with morphology compatible with *Leptospira* under 20× magnification of a fluorescent microscope. Inset is the same at 40× magnification.

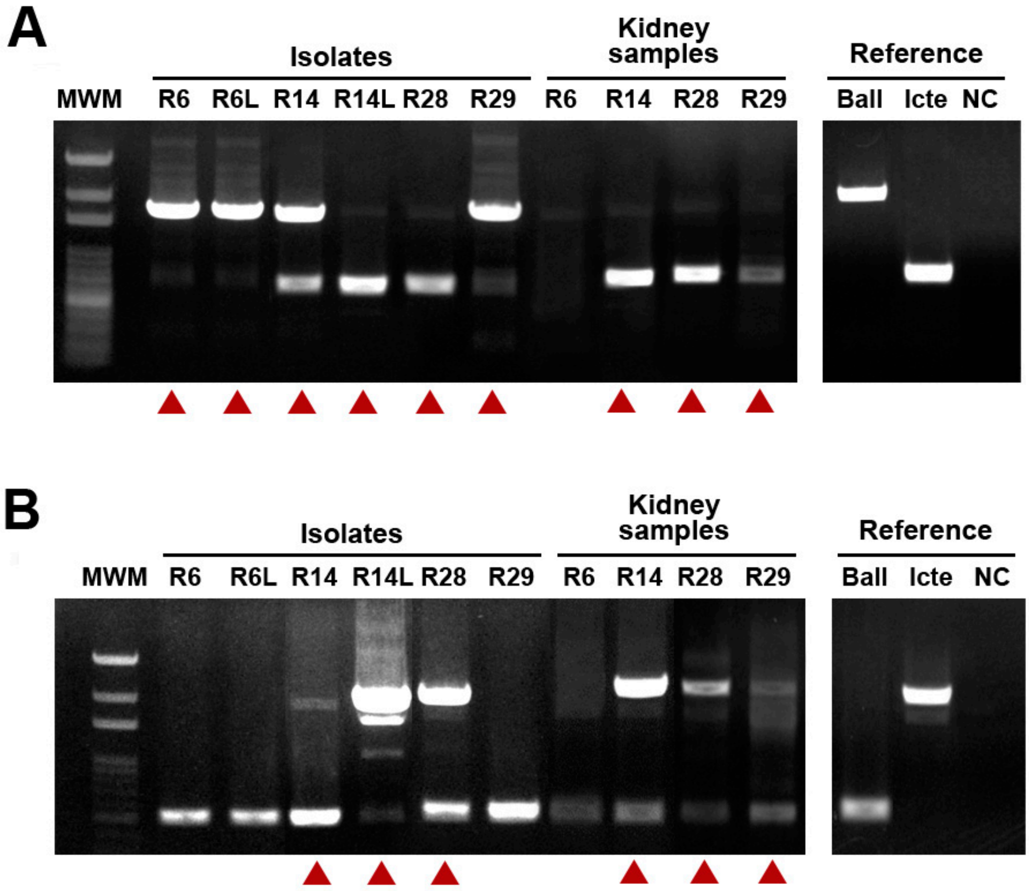

Figure S3. PCR amplification of potential species-specific sequences from isolates and original kidney samples. Figure shows agarose gels stained with ethidium bromide, ran under the same conditions for amplification products of **(A)** IS1500 and **(B)** IS1533. Reference strains for *L. borgpetersenii* serogroup Ballum (Ball) and *L. interrogans* serogroup Icterohaemorrhagiae (Icte) are also included, along with a negative control (NC). Samples with evidence of mixed infection are indicated with red triangles. These are samples for which a band corresponding to a different species was observed alone or in addition to the one for the expected species.
